# Some Phases of Pyorrhea Alveolaris and Its Treatment

**Published:** 1903-10-15

**Authors:** Geo. W. Cook

**Affiliations:** Chicago, Ills.


### DENTAL REGISTER.

Vol. LVII. OCTOBER 15, 1903. No. 10.

### SOME PHASES OF PYORRHEA ALVEOLARIS AND ITS TREATMENT

BY GEO. W. COOK, B.S., D.D.S., CHICAGO, ILLS.

Read before the Michigan State Dental Association, July 8, 1903.

It would be interesting as well as profitable to give the conceptions heretofore held concerning infectious diseases, but such a history would be impossible in one brief paper; suffice it to say that all diseases of which we have any definite knowledge are looked upon as micro-parasitic processes.

In local disease processes there is one of three changes always taking place in the tissue cells, either a regenerative, degenerative or necrotic change. In pyorrhea alveolaris we have the regenerative and the necrotic changes. The degenerative process is the one most commonly observed in certain phases of pyorrhea alveolaris. However, this particular phase can not easily be separated from both the other changes that take place in the tissues, namely regeneration and necrosis.

Since all local pathological changes in tissues are believed to be due to some external exciting cause, consequently, pyorrhea alveolaris must necessarily be classed as springing from the action of bacteria and their products.

Pyorrhea alveolaris belongs to that class of diseases to which only a limited number of individuals seem to be predisposed, as is the case with rheumatism, gout, actinomycosis, cancer, etc., all of which are looked upon as belonging to a class of specific infectious diseases. The question might be asked, why do not all diseases attack all mankind, seeing that they are everywhere disseminated? This question can only be answered by stating that micro-organism, are not always constant in their pathogenic properties; neither are the tissues and the cells of the body at all times endowed with the same predisposition to the exciting cause of the disease, which may be, or is looked upon as being, some external agent. In the mouth germs live in an environment subject to sudden and extreme changes. The saliva is a solution containing chiefly the electrolytic salts, sodium, potassium and magnesium ions.

Since it has been thoroughly demonstrated that the life of the protoplasm, as exhibited in its irritability, depends upon the proper qualitative and quantitative relations of these ions in the environing solutions, it is easy to understand how a change in the composition of the salts of saliva will affect the physiological phenomena of bacteria. Should the change in the saliva be of such a nature as to lessen the irritability of bacteria, their disease-producing qualities would necessarily be diminished. The ionic constituents of the environing fluids of the protoplasm become changed in the tissues of the body in such a way that the resisting power to bacterial invasion may become lessened; consequently, the irritating quality of the bacteria and their products remaining the same, they may become irritating factors, and, after a considerable length of time, bring about that peculiar, slow, degenerative process that is so frequently observed in pyorrhea alveolaris, where we do not have that active necrotic change that is sometimes observed in the formation of pus.

In this slow, degenerative process the tissue must come in contact with a poison, and the primary reaction being that of increased irritability the increased activity of the affected tissue is of a protective tendency. Such poisons always give rise to the so-called inflammatory phenomenon, namely, a gathering of the leucocytes and tissue cells to the affected area or toward the offending agent. If the offending agent is not of sufficient strength to destroy the tissue elements as fast as they accumulate the agent is soon destroyed or rendered ineffective. Here the internal resistance is greater than the external cause (bacteria).

It must be considered, however, that inflammation and degeneration completely break down and the nuclei of the cells become suspended in serum, and with the dead tissue cells form a necrotic mass, therefore, necrosis is not a process but a result of inflammation and degeneration. The term applied to the process previous to the complete breaking down of the tissues is known as necrobiosis; which means a reparative tendency on the part of the living cells and a struggle on part of the offending agent to overcome the internal resistance of the physiological forces of the tissues of the body.

Up to the present time the study of bacterial forms and their classification has been in accordance with the morphology of these unicellular forms, losing sight almost entirely of the chemical factors in the causation of bacterial diseases, a condition that can no longer be ignored, for it is a well recognized fact that many of the disease symptoms that make their appearance in the higher animal organisms are due to the chemical activity of the bacterial cell, and in the majority of instances these general symptoms arise from some local bacterial process. This is well illustrated in the diseases known as diphtheria and tetanus. In both instances we have a local degenerative process in which the bacteria themselves seldom, if ever, enter the general circulation, but during their activity they form an intercellular bacterial substance that has an elective affinity for the general nerve centers, which bring about certain well defined clinical symptoms of these diseases.

In pyorrhea alveolaris we have a local degenerative process in which there is constantly present numerous bacteria, and in some instances it may be necessary for there to be present more than one form in order that the diseased condition manifest its true type of meaning, which is pus in the pockets. This term is known as symbiosis, a condition in which there is more than one bacterium present during the general activity of the disease.

It is a well established fact, however, that the pathogenic bacteria has the power to incite disease, and some of them have the ability to lead a saprophytic existence with other organisms. Consequently, all the rules laid down by Koch cannot be definitely complied with in cases of all diseases. Victor C. Vaughan has said that every germ causing disease in man does so by its chemical product. In pyorrhea alveolaris we have a local degenerative process of certain elements which always takes place in a definite kind of structure, and when this tissue has been removed, as for instance, the tooth, complete recovery from the disease takes place, and most of the constitutional symptoms disappear. Therefore, it is fair to presume that pyorrhea alveolaris is a specific disease process of local origin, and all general symptoms are the result of the absorption of products of the local process.

It has been my privilege to study a number of these cases in which there was every evidence of a general constitutional disturbance. Taking cultures from the pyorrhea pockets and allowing them to grow from twenty-four to forty-eight hours in various forms of culture medias, and especially in one containing no proteid substance; and filtering out the bacteria and injecting from day to day small quantities of this culture media into animals, I have been able to bring about general constitutional symptoms of languor, and the animals eventually become emaciated and in some instances die. Some would live for a considerable time, and when the injections were stopped they would in a very short time return to their general diet and soon recover. Controlled animals were used and injected with the same quantity of the solutions in which no bacteria had been grown. This was done in order to determine if the solution as prepared for the food media for bacteria had a deleterious effect upon the animals. None of the controls were affected by the solution.

It would be quite out of place for me at this time to attempt to discuss the chemistry of the media in which these micro-organisms grew; suffice it to say that the culture media injected into animals were rendered neutral before injection.

The evidence here obtained is sufficient to demonstrate conclusively that many of the symptoms of a constitutional nature that are manifested in pyorrhea alveolaris are due, in most instances, to the pyorrheal infection. There may, however, be some conditions where the constitutional manifestations are established before the typical and well-defined symptoms of the local pathological condition has bcome well defined; for instance, there may be poisonous substances formed in the intestines during external digestion and which exert their influence after being absorbed into the body through the intestinal wall, thus bringing about the predisposing factors to pyorrhea alveolaris.

I have elsewhere discussed at considerable length those conditions that seem to be very important factors in the etiology of pyorrhea alveolaris, and when we have clearly in mind the various factors that enter into the etiology of this morbid process, the treatment must therefore be based upon our conception of the true causes that enter into this condition.

I may here speak of the treatment of that phase of pyorrhea in which we have but little pus formed, but where there is a slow degeneration forming deep pockets along the side of the root in which there is firm attachment of the peridental membrane, possibly a half or two-thirds around the root. In such cases the disease has not been observed by the patients and they have suffered but little inconvenience, consequently, there is a number of teeth around which the pockets have been established without any knowledge or inconvenience to the patient. On close inquiry it will be learned that the patient has suffered from no constitutional difficulties and will claim that the appetite is fairly good and digestion also good. These persons are many times of a robust nature and are commonly supposed to be in a healthy condition. In the more advanced stages of pyorrhea in these cases it will be found that the disease is not so controllable in winter as in summer, especially among that class of patients as are confined to indoor and sedentary life; the appetite and digestion is usually good, but in most cases there is a lack of proper activity of alimentary digestion and elimination.

In such cases there is an accumulation of both saprophytic and fermentative bacteria in the intestinal tract; these agents being much more active in the process of breaking up certain foods, like the carbohydrates which are essentially digested in the small intestines. The energy that under ordinary circumstances is extracted by the small intestine from carbohydrate food is materially increased by certain forms of bacteria that may pass down through the stomach and into the small intestines. The albumenous digestion may likewise meet the same fate by being thus converted into a substance out of which the nutrition of the body has been robbed of some of the most essential and important substance for keeping up the physiological equilibrium of the body. All food media may be interfered with as to render it quite invaluable to the body nutrition, and in this way are brought about many of the forms of predisposition to local infection.

I have elsewhere called attention to these various forms of interference with the physiology of digestion and nutrition, and how it may be used as a means of bringing about predisposition and immunity. Suffice it to say here that the various means of changing the body from extreme immunity to a state of susceptibility are so varied that time and space will not permit us even alluding to the many factors, in some cases it is possible to determine all and others only as many as is consistent with the possibilities of the case in hand. It is not possible to determine the many factors that enter into the typical physiological functions of the body, and still less the many things that may interfere with the functions of the various organs and tissues.

The local conditions, such as the extent and number of teeth involved with this slow degeneration of the peridental membrane and its adjacent structures is of the very first importance. Is there pus, determine so far as possible the comparative quantity in the twenty-four hours; the extent of the deposites on the roots of the teeth, if they should extend, for instance, two-thirds the length of the root with a loss of considerable alveolar process. In such cases it is possible under favorable constitutional conditions to bring the teeth into a healthy state and have these remain so for an indefinite period. One of the important processes in the treatment of such conditions is the removal of the deposits. If they happen to be of a soft, gelatinous consistency their removal is of course very simple, but in cases where the peridental membrane is diseased far beyond the extent of the pocket it would be necessary to lacerate and break up this pathologic structure. This can be done without any special pain, by the use of a local anesthetic (and for that purpose I have found nothing that answered better than acestoria). As an agent for disinfecting in these particular cases there is nothing better than some of the organic acids. Lactic acid is used by many with good results. It is non-escharotic and penetrates deeply into the surrounding tissue. I have incorporated in lactic acid some of the metals making a lactate of silver and copper, and I have come to the conclusion that the lactate of copper is more beneficial than the pure lactic acid. The number of applications of any of these agents depends upon the conditions and the extent of the infected area. Sometimes it is necessary after the deposits have been thoroughly removed to go into the parts and set up an acute inflammation by mechanical means in order that the tissues may become actively hyperemic; in such cases there has been established an active local leucocytosis, and this brings about regenerative activity.

Another phase of pyorrhea that is very closely allied locally to the conditions just described is where there is considerable deposit on the root of the tooth, very firmly attached and of the consistency of a hard, calcareous mass. In such conditions there is usually more extended necrotic condition of the tissue; there is usually a bluish tinge at the margin of the gums, which sometimes will be observed to follow almost the length of the root. It frequently happens that it is a difficult matter to get an instrument into the pocket, especially one that you can operate for the removal of the deposits. In such cases it may be necessary to inject a local anesthetic and split the gum the full length of the blue line, removing all the deposits and dressing the case surgically for a few days and then allow the gum to heal up. In these cases, as a rule, the disease process is confined, in the early stages, to a very few teeth, there sometimes being but one tooth involved. If it is possible to get into the pockets without splitting the gum the deposits are removed and a line of treatment that has given the best success in my hands is used. After removal of deposits I used a preparation known as phenol sulfonic acid. This preparation is made by combining equal parts of crude carbolic acid and crude sulphuric acid. It is a very active antiseptic and has the peculiar power of penetrating deeply into the tissue that is undergoing the degenerative process, especially in cases where there is destruction of bony tissue. It has a chemical action on the deposits and many times assists in loosening them so that their removal is much more easily effected. Two or three applications of this agent is sufficient after the deposits have been surgically removed. During the operation of removing the deposits every surgical precaution should be used, avoiding as far as possible the use of any agent that is an oxidizer or an escharotic, both of which leave the tissues an easy prey to the action of the micro-organism. A warm salt solution should be kept at hand, and during the operation the pockets should be vigorously irrigated with this solution.

After the operation of removing all deposits has been effectively executed under aseptic conditions and the treatment followed out as herein set forth, upon such teeth where the pathological degenerative process has been extended over two-thirds the length of the root and more than one-half of the circumference of the root, the restoration of the tooth to a useful condition is very hopeful; but that predisposition of the individual must also be removed, otherwise local treatment can only be temporary. This predisposition may to a very large extent be a local one, in other words, we recognize that the provisions of food for the certain production of bacterial energy rests very largely with the bacterial environments.

A normal saliva under certain circumstances, mixed and mingled as it is with other nutritional properties, may be sufficient to increase the pathogenic properties of a bacterium to its very highest efficiency, and circumstances making it possible for micro-organisms to find a lodgment under the free margins of the gum, they set up a degenerative process of the tissue cells with which they come in direct contact, and eventually establish the well-defined symptoms that accompany pyorrhea, very few of which are ever observed in the very early stages of the disease, unless, there has been some long organic disturbance of the body. In such cases they are only predisposing factors, and it is possible for them to have but little influence on the real virulent properties of that micro-organism which is constantly present in this disease. On the other hand an organic disease may change the saliva in its electrolytic properties in a way that it may act favorably or unfavorably upon the protoplasmic structure of the micro-organisms present in the oral cavity. The biological factors that enter into all disease processes is at the present time in more or less obscurity, and especially those diseases where there is present local as well as general manifestations.

At the present time the biological phenonema of pyorrhea alveolaris rests upon two factors: first, that it exists in the animal economy as a predisposition which may be inherited or acquired, and second, that bacteria are the exciting cause. When these factors are taken into consideration and the exact bearing that the one has to the other, both from a clinical and scientific standpoint, and the treatment followed out in accordance with the physiological lesion, pyorrhea is amenable to treatment under favorable circumstances.

DISCUSSION.

DR. J. TAFT: This is a wide-spread disease, and any paper on this subject will command attention. The one to which we have just listened will bear much thought and study as it attempts to call our attention to the etiology as well as the more practical therapeutics of this interesting disease. A large proportion of the teeth once affected with this disease are doomed. It begins in an insidious way attacking one tooth and then another until by and by the entire jaw is affected. Sometimes it appears simultaneously by attacking all the teeth and running its course rapidly and with disastrous consequences. Usually the teeth loosen one by one and take on inflammatory processes which hasten their extraction. Treatment in some cases seems futile, and no known treatment is a specific in all cases. The pathology of the disease is so varied that no one could give it in so short a time. The essayist has given one aspect lucidly, but it would require a volume to give the complete history and treatment of every form. In my judgment constitutional conditions play a very important part in the development and control of it. Patients even show variations in the more localized incidents. It does not run the same course with all patients, and all patients do not react similarly to the similar treatment.

Local factors are always present, either of a mechanical, chemical or microbic form. It is a curious fact that the disease not always attacks the entire tooth environment, but, as cited by the essayist, it may extend laterally over a small area of the root, but deeply towards the apex. Why this should be it is not always easy to determine. It may be due to the kind of infection present or to some aberration in embryonic development of the peridental structures rendering them more susceptible in certain areas. At times we meet cases in which the process is painless, and still others in which there is excessive sensitiveness of the roots and more or less neuralgic or inflammatory pains.

My own opinion is that it is essentially a disease of the peridental membrane rather than of the gum tissues. There is almost always a degeneration of the peridental membrane, while the associated gum may retain an unusually normal condition, but of course as the disease progresses the gum inevitably becomes involved, although the overlying mucous membrane is seldom seriously involved. A proof that the disease is peridental is that when the teeth are extracted healing occurs without delay. The treatment will always vary with the manifestations. If there is suppuration or necrosis, disinfection is indicated both of a surgical and chemical character.

Dr. Oakman: We should know the pathological conditions that exist in order that we may apply proper treatment, but it is not always necessary that the etiologic history be known.

I have had excellent results from the use of lactic acid, especially in necrotic conditions and where an escharotic remedy is not contra-indicated. It however usually causes considerable absorption, as it destroys and shrinks the tissues.

In scaling and operative procedures I do not like to destroy the gum septa, but see no objection to making the incision over the root as recommended by the essayist. This would not be likely to prove detrimental by causing absorption and would readily heal. Use small, thin instruments so as not to mangle the gum unduly.

I have a patient who is a hard drinker, and when he came to me twenty-seven of his teeth were badly affected with considerable gum recession. The patient was urged to moderate in his bibulous habits, and the treatment undertaken. The first operations were made to remove the deposits, and it became necessary to cut through the gum substantially as the essayist recommends. The teeth were thoroughly cleansed of all deposits after several sittings. After three months' treatment the gums are in good condition and the patient has comfort where before he suffered much pain and discomfort. It has required constant care on both my part and the patient's.

My method is to first thoroughly cleanse the teeth surgically and mechanically, then I disinfect them usually with hot solution of chlorotone, which is a good anodyne antiseptic used hot. I make a supersaturated solution in a test tube and use it while still as hot as the patient can stand it. It sterilizes the operative field and eases the pain so that further sealing may be done comfortably if necessary, and it leaves a stimulating influence as it is absorbed. If the gums are congested at the first operation I bleed them freely. In addition to local treatments which will be suggested by the symptoms which develop, I instruct the patient to massage the gums two or three times daily with the fingers or with the brush when cleansing the teeth. If there is an acid reaction to the secretions of the mouth I would use some good alkaline antiseptic. If the teeth are affected, it is desirable that they be put into a splint to hold them in their normal position with the least amount of change from the force of mastication, which is likely to prolong the irritation and may result in the loss of the teeth.

Dr. Warboys: I find it very desirable to control the hemorrhage as well as the pain in any kind of surgical work upon the teeth. I have found nothing better for this purpose than a solution of cocain in adrenalin, about a two percent solution of cocain. If it is necessary to bleed the gums I wait until after much cleansing is done, and then if it is still called for make liberal incisions so as to keep up the withdrawal of blood for several minutes. My method is to first thoroughly clean the teeth and then disinfect the surrounding or implicated areas, and then apply antiseptic or curative treatment as indicated.

Dr. E. B. Lodge, of Cleveland, O.: There are two treatments that have not been mentioned that I have used with good success. One is the decomposition of remedial agents at the seat of the disease by electrolysis. This may be accomplished by packing the pockets first with crystalline substances and decomposing them with an electrode, or by use of an electrode made of a metal or alloy which will be decomposed by the current or chemical generated at the point of contact with the diseased structures. The other is the use of the X-rays applied directly to the tissues affected. This of course requires special apparatus and skill.

Dr. N. S. Hoff: It is practically impossible for any one to treat all cases of pyorrhea by the same methods and get successful issues. There are not only wide differences as to cause and conditions, including systemic and habitual circumstances which are never the same in two cases. While it is possible to conclude that the disease has certain phenomena that are more or less constant and common, there will be sufficient differences in individual cases to make it necessary to modify any course of treatment to meet these differences. We must be able to diagnose the condition met and determine as far as practicable its peculiarity before we can intelligently apply treatment. Then again, any treatment will necessarily have to be watched and varied to meet the different stages as they develop. Too long continuation of a treatment of a caustic or alkaline nature may result in unecessary loss of the soft tissues, and, on the other hand, the treatment be unnecessarily prolonged because the remedy has not been applied in sufficient power to accomplish the required action. The manifestations of this disease are so manifold that great skill and vigilance are essential to overcome it. If we can not determine its etiology, we can note the symptoms, and it will be well if we follow these carefully and apply our remedies with intelligent discrimination.

Dr. Geo. W. Cook: The study of the etiology especially, and the pathology also of this disease is a much larger subject than most of us imagine. I have given to it a large part of my study hours for the past seven years, and I do not yet comprehend it, although I think I am in sight of some phases of it at least and I expect from these in time to get a more definite knowledge of its character, and then its treatment will necessarily follow.

The oral structures are subject to the same rules which obtain with other tissues. In some mouths, even though there is great provocation to infection, it never occurs; while in others where great care is exercised to protect the tissues from infection by various sterilizing methods, the disease will run a rapid course. In these cases we can only conclude that there is local predisposition. This of course we can not change by any form of local treatment alone. Possibly in such cases a complete systemic renovation is required. In addition to our efforts to eliminate all extraneous or local causes the family physician must be called in for complete systemic treatment, including a general toning up of the nutritive processes.

We should of course exercise great judgment in the application of remedies. I sometimes use lactic acid effectively in cases of acute inflammation, but its action may serve to accentuate the disease. Phenol-sulphonic acid should never be used where there is irritable tissue unless you want to destroy it. It is an excellent remedy where there is necrotic structure to break down and disinfect.

It is not likely that the peridental membrane, the alveolar process or the gum tissue is ever developed to its normal condition. The gum may to some extent in favorable locations be made to grow upon the root, but it is repair tissue and has no great endurance under the fierce attacks made by the usual functional activities of the mouth.

